# Common maternal health problems among Australian-born and migrant women: A prospective cohort study

**DOI:** 10.1371/journal.pone.0211685

**Published:** 2019-02-11

**Authors:** Tharanga Navodani, Deirdre Gartland, Stephanie J. Brown, Elisha Riggs, Jane Yelland

**Affiliations:** 1 Intergenerational Health Group, Murdoch Children’s Research Institute, Parkville, Victoria, Australia; 2 Ministry of Health, Colombo, Sri Lanka; 3 Department of Paediatrics, University of Melbourne, Parkville, Victoria, Australia; 4 Department General Practice and Primary Health Care Academic Centre, University of Melbourne, Parkville, Victoria, Australia; Xi’an Jiaotong University School of Medicine, CHINA

## Abstract

**Background:**

Migrant women of non-English speaking background make up an increasing proportion of women giving birth in high income countries, such as Australia, Canada and the United Kingdom. The aim of this study was to assess the prevalence of common physical and psychosocial health problems during pregnancy and up to 18 months postpartum among migrant women of non-English speaking background compared to Australian-born women.

**Methods:**

Prospective pregnancy cohort study of 1507 nulliparous women. Women completed self-administered questionnaires or telephone interviews in early and late pregnancy and at 3, 6, 9, 12 and 18 months postpartum. Standardised instruments were used to assess incontinence, depressive symptoms and intimate partner violence.

**Findings:**

Migrant women of non-English speaking background (n = 243) and Australian-born mothers (n = 1115) reported a similar pattern of physical health problems during pregnancy and postpartum. The most common physical health problems were: exhaustion, back pain, constipation and urinary incontinence. Around one in six Australian-born women (16.9%) and more than one in four migrant women (22.5%) experienced intimate partner abuse in the first 12 months postpartum. Compared to Australian-born women, migrant women were more likely to report depressive symptoms at 12 and 18 months postpartum.

**Conclusion:**

Physical and mental health problems are common among women of non-English speaking background and Australian-born women, and frequently persist up to 18 months postpartum. Migrant women experience a higher burden of postpartum depressive symptoms and intimate partner violence, and may face additional challenges accessing appropriate care and support.

## Introduction

Pregnancy and childbirth are major life events that place complex demands on women’s physical and psychological health and wellbeing [1]The physical and emotional changes associated with pregnancy and early motherhood can be especially challenging for migrant women having a baby in a new country[2] One third of all women giving birth in Australia are migrants, the majority coming from a country where English is not the main language or official language [3]. Migrant women of non-English speaking background settling in high income countries are known to experience multiple and varied barriers accessing perinatal health care [4–7]. A comparative review of studies conducted in five high income countries found that communication problems, perceived discrimination and lack of familiarity with health care systems had a negative impact on migrant women’s experiences of care [8].

The circumstances of migration and settlement can accentuate women’s physical, social and emotional vulnerability, contributing to poor health during pregnancy, adverse birth outcomes and poorer postnatal mental health. A systematic review of studies examining migrant women’s access to health care in countries of settlement found evidence of: higher morbidity during pregnancy, higher incidence of stillbirth and early neonatal death, increased risk of maternal death, and higher incidence of postpartum depression comparing migrant and native born women. [6]. Among the studies cited in this review are several cross-sectional studies documenting poorer postnatal mental health among migrant women ([4, 9, 10] Other studies conducted in the first 6 months postpartum report similar findings [2, 11]. However, few studies have reported on the prevalence of common maternal postpartum physical morbidities among migrant women. Nor have studies examined the physical or psychological health and wellbeing of migrant mothers beyond the first few months after childbirth.

Drawing on data collected in a large Australian prospective pregnancy cohort study, the aim of this paper is to assess the prevalence and natural history of common physical, emotional and social health problems during pregnancy and up to 18 months postpartum among migrant women of non-English speaking background compared to Australian-born women. We hypothesised that migrant women of non-English speaking backgrounds would experience greater morbidity than Australian-born women.

## Materials and methods

### Study participants

The Maternal Health Study is a prospective, pregnancy cohort study designed to investigate the health and wellbeing of first time mothers during pregnancy and the first 18 months after childbirth. The primary aim of the study was to improve understanding of the natural history (onset, persistence, severity) of common maternal physical and psychological health problems during pregnancy and after childbirth. The study also aimed to investigate the prevalence of intimate partner violence in the first 12 months after childbirth.

Women booking to give birth in six metropolitan public maternity hospitals in Melbourne, Australia were recruited to the study over a two-year period. To be eligible for the study women needed to be nulliparous, have a gestation of 24 weeks of pregnancy or less (according to the date of last menstrual period or ultrasound), be 18 years of age or above, and have sufficient proficiency in English to complete written questionnaires and telephone interviews.

The participants were recruited by a mailed invitation facilitated by the six participating hospitals. The invitation package included a copy of the baseline questionnaire and study consent form. This was followed up by a single reminder postcard. At the time of recruitment women were informed about the frequent follow up, long term commitment and the personal and potentially intrusive nature of questions about common maternal health issues such as incontinence and sexual health issues. Women were invited to return the questionnaire, signed consent form and contact information in a reply paid envelope provided in the invitation package.

Participants were invited to complete follow-up questionnaires at 3, 6, 12 and 18 months postpartum, and computer-assisted telephone interviews (CATI) at 30–32 weeks of pregnancy and at 9 months postpartum.

### Measures

Socio-demographic data including maternal country of birth, date of birth, relationship status and income were collected in the baseline questionnaire in early pregnancy.

Common physical and psychological problems: Maternal physical and psychological health problems were assessed in the baseline questionnaire and at each follow-up using a series of single item questions about specific health issues, including extreme tiredness or exhaustion, anxiety symptoms, severe headaches or migraines, breast problems, back pain, haemorrhoids and constipation. Women were able to choose between four response categories (‘never’, ‘rarely’, ‘occasionally’ and ‘often’), and were classified as having a health issue if they responded ‘occasionally’ or ‘often’.

Incontinence: Urinary incontinence was assessed using the Incontinence Severity Index developed by Sandvik and colleagues, which has been validated in Australian, Scandinavian and United Kingdom populations.[12, 13] Women who reported leakage of urine at least once a month were categorised as having urinary incontinence. Assessment of bowel symptoms was based on questions adapted from an Australian community prevalence study [14]. Women who reported leakage of liquid or solid stool at least once in a three month period were categorised as having faecal incontinence. Constipation was defined as “opening bowels only twice a week or less, or needing to push and strain to open bowel more than every fourth time of bowel opening”.

Depression: Depressive symptoms were assessed using the Edinburgh Postnatal Depression Scale (EPDS), a ten item self-report scale originally developed to identify depressive symptoms in the postnatal period [15], but also validated for use in pregnancy [16]. The recommended standard cut-off score of 13 or above was used to define women as having probable major depression [15, 16].

Relationship problems and intimate partner abuse: At 12 months postpartum women were asked about relationship problems with their partner (or ex-partner) over the preceding three months, with four response items *never*, *rarely*, *occasionally*, *and often*. Women who responded ‘occasionally’ or ‘often’ were categorised as having relationship problems. Intimate partner abuse was assessed using the short version of the Composite Abuse Scale (CAS) which comprises 18 items of actions by an intimate partner or ex-partner that constitute emotional or physical abuse. For each item women were asked how frequently a specific behaviour had happened to them during the previous 12 months (i.e. since the birth of their first baby). Response options included *never*, *only once*, *several times*, *once per month*, *once per week* and *daily*. Physical abuse was defined as a score ≥ 1 on the physical abuse scale and emotional abuse as a score of ≥ 3 on the emotional abuse scale [17].

### Data analysis

Analyses presented in the paper compare outcomes for Australian-born women and migrant women of non-English speaking background (NESB). Women born overseas in countries where English is not the national language (e.g. countries in the Middle East, Asia, Africa, parts of Europe) were categorised as ‘migrant women’. Data from consecutive population-based surveys of women giving birth in Victoria in 1989, 1993, 2000 and 2007 show that the health and health care experiences of migrant women from English speaking countries such as New Zealand and the United Kingdom are similar to those of Australian-born women [1, 18]. Therefore women born overseas from English speaking countries were excluded from analyses presented in the paper.

Data were analysed using STATA version 14.0. Univariable logistic regression was used to explore differences in the socio-demographic characteristics, physical health, mental health and intimate partner relationships of migrant women compared to Australian-born women. Multivariable logistic regression was used to obtain a clearer understanding of the differences in physical and mental health for migrant women compared to Australian born women, adjusting for maternal age and mode of birth due to established associations with maternal health, and sociodemographic factors that differed significantly between migrant and Australian-born women in univariable analyses.

### Ethics

Ethical approval for the study was granted by institutional ethics committees at the participating hospitals and the ethics committees of La Trobe University and the Royal Children’s Hospital, Melbourne.

## Findings

### Participant characteristics

A total of 1507 eligible women enrolled in the study, with a mean gestation at enrolment of 15.0 weeks (SD = 3.1, range 6–24 weeks). The majority of women in the study were Australian-born (n = 1115, 74.4%), 141 (9.4%) were migrant women from English speaking countries such as the UK and New Zealand, and 243 (16.2%) were overseas born of non-English speaking background. The representativeness of the cohort was assessed by comparing participant characteristics to routinely collected data for births in the study recruitment period. This showed that participants were largely representative in relation to method of birth, infant birthweight and gestation, but younger women (18–24 years, 15.5% versus 29.9%) and women of non-English speaking background (16.2% versus 21.0%) were under-represented. It was not possible to determine a precise overall response rate as the participating hospitals were unable to identify the proportion of ineligible women (e.g. multiparous women) who received an invitation. We conservatively estimate that the response rate was in the range of 28 to 31%, but it may have been higher. Follow-up response rates were: 96% in late pregnancy (1454/1507), 95% at 3 months (1431/1507), 93% at 6 months (1400/1507), 90% at 12 months (1357/1507), 88% at 18 months (1327/1507).

Migrant women from English speaking countries (n = 141) and women who did not report country of birth (n = 8) were excluded from analyses presented in the paper, leaving a sample of 1358 women. Table 1 presents the socio-demographic characteristics of Australian-born and migrant women of non-English speaking background (from here on referred to as migrant women) at enrolment in early pregnancy. Over half of the migrant women (55%) were from Asia, with India (n = 26) and Sri Lanka (n = 21) the most common Asian countries of origin. Other migrant women came from European (26%), South American (9.9%) and African (8.6%) countries. As shown in Table 1, migrant women were more likely to be married, with significantly lower odds of being in a de facto relationship (OR = 0.3, 95%CI 0.2–0.5) or being divorced/single/separated (OR = 0.1, 95%CI 0.0–0.4) compared with Australian-born women. They were also more likely to have a university degree and less likely to be in paid employment. There were no significant differences in maternal age or method of birth. Just under half of the Australian-born and migrant women had a spontaneous vaginal birth (49% and 48% respectively). One in five Australian-born and migrant women had an operative vaginal birth (21.2% and 22.2% respectively), while 30.0% of Australian-born women and 30.4% of migrant women had a caesarean section.

**Table 1:** Socio-demographic characteristics of Australian-born and migrant women of non-English speaking background (n = 1358).

	Australian-born women n = 1115		Migrant women n = 243		OR (95% CI)^a^	Pearson Chi^2^_(df)_ p-value
	n	%	n	%		
**Maternal age** (years)						
18–24	193	17.3	32	13.2	0.8 (0.5,1.2)	Χ^2^_(4)_ = 5.00
25–29	363	32.6	93	38.3	1.2 (0.8,1.6)	p = 0.286
30–34	396	35.5	87	35.8	1.0 (ref)	
35–39	140	12.6	25	10.3	0.8 (0.5,1.3)	
40+	23	2.1	6	2.5	1.2 (0.5,3.0)	
**Relationship status**						
Married	626	56.1	196	80.7	1.0 (ref)	Χ^2^_(2)_ = 51.76
De facto (Living with partner)	427	38.3	45	18.5	**0.3 (0.2,0.5)**	p < 0.001
Divorced/Separated/Single	62	5.6	2	0.8	**0.1 (0.0,0.4)**	
**Educational attainment**						
University degree	463	41.7	143	59.3	1.0 (ref)	Χ^2^_(3)_ = 28.50
Certificate/Diploma	299	26.9	55	22.8	**0.6 (0.4,0.8)**	p < 0.001
Year 12	226	20.4	32	13.3	**0.5 (0.3,0.7)**	
<Year 12	122	11.0	11	4.6	**0.3 (0.2,0.6)**	
**Total income** (Aus $)						
≤ 20,000	161	14.5	50	22.4	**1.6 (1.0,2.3)**	Χ^2^_(3)_ = 46.41
20,001–40,000	400	36.1	80	35.9	1.0 (ref)	p < 0.001
40,001–60,000	346	31.3	42	18.8	**0.6 (0.4,0.9)**	
> 60,000	140	12.6	17	7.6	0.6 (0.3,1.1)	
**Pension as main income**						
No	95	8.6	22	9.3	1.0 (ref)	Χ^2^_(1)_ = 0.125
Yes	1009	91.4	214	90.7	1.1 (0.7,1.8)	p = 0.723
**Employment**						
Paid work	943	86.1	155	64.3	1.0 (ref)	Χ^2^_(4)_ = 76.98
Unpaid work	39	3.6	30	12.4	**4.7 (2.8,7.8)**	p < 0.001
Study	21	1.9	20	8.3	**5.8 (3.1,10.9)**	
Unemployed	39	3.6	17	7.1	**2.7 (1.5,4.8)**	
Not able work	53	4.8	19	7.9	**2.2 (1.3,3.8)**	

^a^ Odds ratios (OR) refer to the odds of each category (characteristic) occuring among migrant women compared with Australian-born women, in relation to the reference category (ref).

### Common physical health problems

Table 2 shows the prevalence of a range of health problems experienced by Australian-born and migrant women during pregnancy and up to 18 months postpartum. The most common physical health problems were: extreme tiredness/exhaustion, back pain and constipation. Exhaustion was most common during early pregnancy, with more than three quarters of women in both groups reporting this symptom at enrolment, decreasing to just under half of women affected in late pregnancy. Back pain showed the reverse pattern; around half of women reporting this health problem in early pregnancy, increasing to over two thirds of women in both groups by late pregnancy. After the birth, exhaustion and back pain were the most common maternal health problems, affecting over 60% of Australian-born and migrant women at each follow-up (with the exception of nine months postpartum when the reported prevalence was somewhat lower in both groups). Haemorrhoids, constipation, and breast problems were most common soon after birth and continued to be experienced by a minority of women at 18 months postpartum. The prevalence of women reporting pelvic pain and/or headaches/migraines appeared to remain fairly stable over time in both groups, while the proportion of women reporting coughs, colds and minor illnesses appeared to increase.

**Table 2:** Prevalence of common physical and psychological health problems during pregnancy and up to 18 months after childbirth.

	Early pregnancy		Late pregnancy		3 months		6 months		9 months		12 months		18 months	
	Aust.born^a^	Migrant	Aust.born	Migrant	Aust.born	Migrant	Aust.born	Migrant	Aust.born	Migrant	Aust.born	Migrant	Aust.born	Migrant
	n = 1115	n = 243	n = 1081	n = 229	n = 1074	n = 212	n = 1054	n = 202	n = 1048	n = 197	n = 1026	n = 188	n = 997	n = 187
	n (%)	n (%)	n (%)	n (%)	n (%)	n (%)	n (%)	n (%)	n (%)	n (%)	n (%)	n (%)	n (%)	n (%)
Extreme tiredness/exhaustion	984 (88.3)^b^	189 (78.1)	473 (43.8)	79 (34.5)	707 (66.3)	140 (67.3)	629 (59.9)	113 (56.2)	436 (41.6)	81 (41.1)	626 (61.2)	117 (62.6)	702 (70.8)	123 (66.1)
Back pain	508 (45.7)	119 (49.2)	750 (69.4)	171 (74.7)	647 (60.5)	131 (62.7)	620 (58.9)	126 (62.4)	554 (52.9)	109 (55.3)	545 (53.2)	98 (52.4)	547 (55.0)	112 (59.9)
Constipation	493 (44.3)	99 (41.4)	NA^c^	NA	277 (26.0)	80 (38.8)	136 (13.0)	40 (20.1)	99 (9.5)	35 (17.9)	99 (9.7)	23 (12.4)	142 (14.4)	35 (18.9)
Haemorrhiods	85 (7.7)	23 (9.7)	125 (11.6)	38 (16.6)	290 (27.2)	64 (31.1)	181 (17.3)	31 (15.5)	101 (9.7)	24 (12.2)	120 (11.8)	26 (13.9)	150 (15.2)	32 (17.3)
Coughs, colds/other minor illnesses	230 (20.7)	59 (24.4)	192 (17.8)	37 (16.2)	204 (19.2)	45 (21.6)	259 (24.7)	49 (24.5)	249 (23.8)	49 (24.9)	375 (36.7)	61 (32.6)	388 (39.1)	60 (32.4)
Severe headaches or migraines	335 (30.1)	78 (32.2)	99 (9.2)	16 (7.0)	175 (16.4)	48 (23.0)	199 (19.0)	42 (21.0)	156 (14.9)	32 (16.2)	248 (24.4)	41 (22.5)	253 (25.7)	51 (27.7)
Pelvic pain	212 (19.1)	34 (14.2)	203 (18.8)	60 (26.2)	122 (11.4)	31 (15.0)	80 (7.6)	19 (9.5)	105 (10.0)	12 (6.1)	70 (6.9)	10 (5.3)	122 (12.3)	9 (4.9)
Breast problems	NA	NA	NA	NA	402 (37.7)	67 (32.2)	110 (10.5)	13 (6.5)	93 (8.9)	12 (6.1)	73 (7.2)	11 (5.9)	92 (9.3)	5 (2.7)
Anxiety/panic attacks	76 (6.8)	26 (10.8)	104 (9.6)	10 (4.4)	164 (15.4)	34 (16.3)	111 (10.6)	22 (10.9)	87 (8.3)	18 (9.1)	93 (9.1)	15 (8.1)	92 (9.3)	22 (11.8)
Fecal incontinence	50 (4.5)	14 (5.8)	19 (1.8)	9 (3.9)	87 (8.1)	15 (7.1)	74 (7.0)	18 (8.9)	30 (2.9)	5 (2.5)	68 (6.6)	11 (5.9)	50 (5.0)	10 (5.4)
Urinary incontinence^d^	178 (16.1)	47 (19.4)	607 (56.2)	129 (56.3)	308 (28.9)	69 (32.5)	204 (19.7)	46 (23.4)	300 (30.3)	63 (33.9)	207 (23.5)	44 (27.2)	198 (27.7)	46 (31.1)
Depressive symptoms	95 (8.5)	28 (11.7)	NA	NA	75 (7.0)	19 (9.1)	94 (8.9)	26 (13.1)	NA	NA	73 (7.1)	24 (12.8)	87 (8.8)	32 (17.2)

^a^ Aust.born = Australian-born women

^b^ Denominators vary because of missing values

^c^ NA = Not available

^d^ Excludes women pregnant with their second child at time of data collection (i.e. 17 pregnant women at 6 months, 67 at 9 months, 168 at 12 months and 320 at 18 months).

Statistically significant differences (derived from data in Table 2) were identified in late pregnancy where migrant women reported a lower prevalence of tiredness/exhaustion (OR = 0.7, 95% CI 0.5–0.9) and anxiety (OR = 0.4, 95% CI 0.2–0.8), and a higher prevalence of haemorrhoids (OR = 1.5, 95% CI 1.0–2.3) and pelvic pain (OR = 1.5, 95% CI 1.1–2.1). Migrant women also had higher odds of reporting constipation at 3 months (OR = 1.8, 95% CI 1.3–2.5), 6 months (OR = 1.7, 95% CI 1.1–2.5) and 9 months postpartum (OR = 2.1, 95% CI 1.4–3.2).

**Fig 1:**
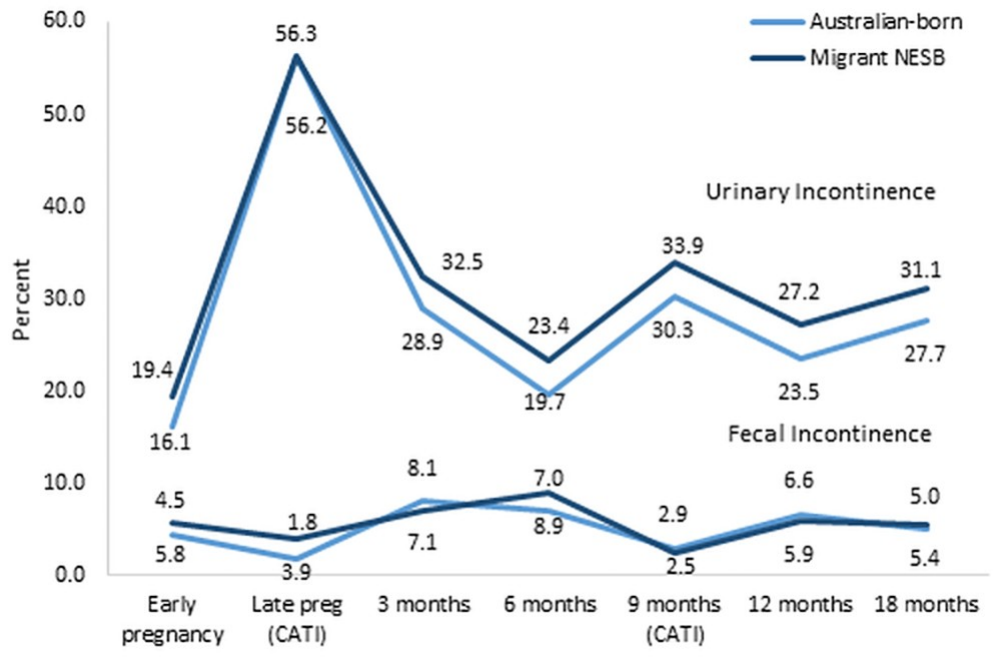
Prevalence of urinary incontinence and faecal incontinence from early pregnancy to 18 months postpartum.

Fig 1 shows the prevalence of urinary incontinence and faecal incontinence during pregnancy and the first 18 months postpartum. In late pregnancy, over half of the Australian-born and migrant women reported urinary incontinence, while less than 4% reported faecal incontinence. More than one in three women reported persisting or recurrent urinary incontinence at 18 months postpartum, compared with about 5% of women who reported persisting or recurrent faecal incontinence. Migrant women generally reported slightly higher prevalence of urinary incontinence, but these differences were not of statistical significance after adjusting for maternal age, method of birth and education level (for example, at 1 year postpartum Adj.OR = 1.2, 95% CI 0.8–1.8). There were no statistically significant differences in the prevalence of faecal incontinence comparing Australian-born and migrant women.

### Maternal mental health

**Fig 2:**
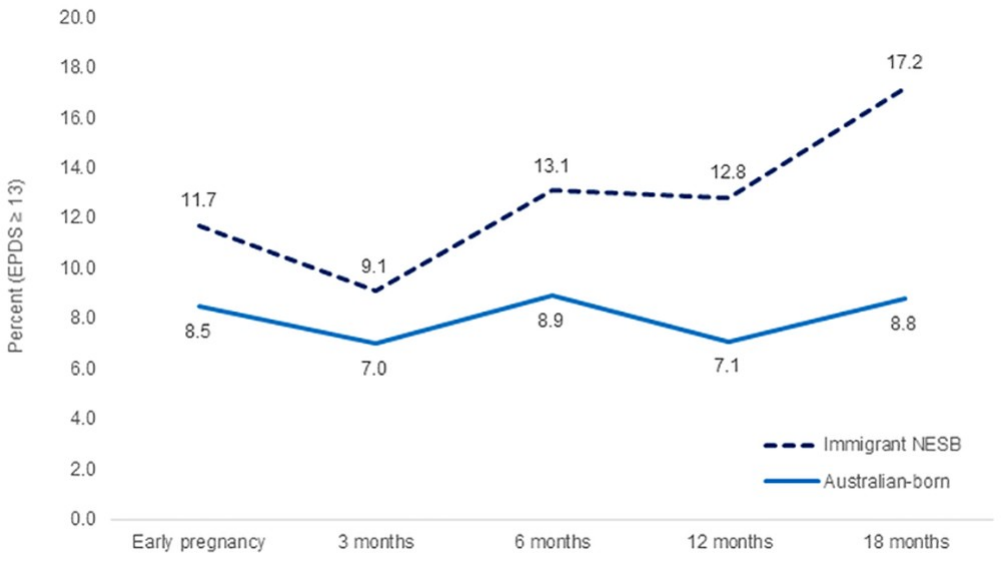
Prevalence of depressive symptoms from early pregnancy to 18 months postpartum.

There were small differences in the proportions of Australian-born and migrant women reporting anxiety symptoms (see Table 2) and/or depressive symptoms (see Fig 2). A higher proportion of migrant women reported anxiety symptoms in early pregnancy (10.8% versus 6.8% of Australian-born women), with the pattern then reversed in late pregnancy (4.4% versus 9.6% of Australian-born women). Around one in ten women in both groups reported anxiety symptoms at each postpartum follow-up. A higher proportion of migrant women reported depressive symptoms (EPDS score ≥ 13) in early pregnancy, and at all follow-up points in the first 18 months postpartum. Differences were statistically significant at 12 (Adj.OR = 2.0, 95% CI 1.2–3.3) and 18 months postpartum (Adj.OR = 2.3, 95% CI 1.4–3.6), after adjusting for maternal age, method of birth and education.

### Relationship problems and intimate partner abuse

Two out of five Australian-born women, and a similar proportion of migrant women reported relationship difficulties in the first 12 months postpartum (Table 3). Around one in six Australian-born women (16.9%) and more than one in four migrant women (22.5%) experienced intimate partner abuse. Compared to Australian-born women, migrant women had significantly raised odds of experiencing intimate partner abuse (Table 3).

**Table 3:** Intimate partner abuse and relationship problems in the first 12 months postpartum (n = 1208).

	Australian-born women n = 1021 n (%)	Migrant women n = 187 n (%)	Odds ratio (95% CI)^c^
**Intimate partner abuse**^a^			
No abuse	848 (83.06)	145 (77.5)	1.0 (ref)
Emotional abuse alone	92 (9.0)	26 (13.9)	**1.7 (1.0, 2.6)**
Physical and emotional abuse	81 (7.9)	16 (8.65)	1.2 (0.7, 2.0)
*Any partner abuse*	*173 (16*.*9)*	*42 (22*.*5)*	*1*.*4 (1*.*0*, *2*.*1)*
**Relationship problems**^b^	465 (42.8)	98 (44.3)	1.1 (0.8,1.4)

^a^ Measured using the Composite Abuse Scale at 12 months postpartum

^b^ Women who reported relationship problems at 3, 6 and/or 12 months postpartum

^c^ Odds ratios (OR) refer to the odds of specific health outcomes among migrant women compared with Australian-born women (as the reference category).

## Discussion

Globally, a sizeable proportion of women giving birth in high income countries such as the United States, Canada, Australia and the United Kingdom are migrants from an increasingly diverse range of countries. The rapid increase in the scale, speed and spread of migration is generating complex challenges for maternity services [19]. There is a growing body of evidence that migrant women of non-English speaking background giving birth in high income countries are at higher risk of a range of adverse birth outcomes, including stillbirth, neonatal mortality and morbidity, and even maternal death [20] However, little is known about the health and well-being of migrant mothers during pregnancy and in the period after childbirth, and how this compares to the health of native-born women.

This is the first multi-centre Australian prospective pregnancy cohort study investigating postpartum physical and psychological health to compare outcomes for Australian-born and migrant mothers of non-English speaking background. The results indicate both similarities, and some differences. Common to both Australian-born and migrant mothers is the extreme exhaustion, characteristic of early motherhood. The study findings reinforce that this experience is not short-lived. A year and a half after the birth of their first baby, over 70% of Australian-born and migrant mothers report extreme exhaustion. Back pain and urinary incontinence stand out as the other highly prevalent and persistent conditions in the first 18 months postpartum, with back pain affecting over 50% of Australian-born and migrant mothers and urinary incontinence reported by almost one in three Australian-born and migrant mothers, a year and a half after the birth of their first child.

Migrant women were more likely to report depressive symptoms compared with Australian-born women, with differences most apparent at 12 and 18 months postpartum. Other studies measuring depressive symptoms at 6 months postpartum have reported similar findings [2, 21]. Bandyopadhyay et al report that migrant women in the months following birth are more likely to report feeling lonely and isolated, and more likely to want practical and emotional support compared to Australian-born women [21]. Other studies examining migrant women’s mental health draw attention to the impact of separation from extended family, low income and marital difficulties [7, 22].

We assessed both relationship difficulties and exposure to intimate partner abuse in the first 12 months postpartum. Intimate partner abuse was measured using the Composite Abuse Scale, an 18 item measure that assesses exposure to both emotional and physical abuse [17]. Both Australian-born women and migrant women experienced high rates of relationship difficulties, with two out of five women reporting relationship problems in the first year after childbirth. Overall, one in six Australian- born mothers and one in five migrant mothers experienced intimate partner abuse. More than half of these women reported emotional abuse alone, as has been found in other studies examining the prevalence and effects of different types of intimate partner violence [23]. Despite the tendency to focus on physical violence, emotional abuse has been shown to have similar detrimental effects on women’s health. It is noteworthy that in our study, migrant mothers had higher odds of experiencing emotional abuse alone, and only slightly raised odds of experiencing physical and emotional abuse (in combination). Isolation from extended family networks and associated challenges of having a baby in a new country are likely to contribute to these findings [24,25].

Health care providers play a pivotal role in assessing and responding to women’s health and social context during and after pregnancy. Yet, women are often reluctant to disclose health symptoms to health professionals, and there may be additional communication barriers for migrant women [6, 23, 24]. Migrant women of non-English speaking background may have low levels of health literacy, limited understanding of the health system, and face difficulties talking about sensitive or personal issues with health professionals even when professional interpreting is available.

### Strengths and limitations

To our knowledge, this is the first propspective population based study to report on the health of migrant and native born women from pregnancy to 18 months postpartum. Further strengths of the study include: frequent follow-up (every three months in the first 12 months postpartum), low attrition, and use of standardised measures to assess common physical and psychological morbidities, and exposure to intimate partner violence.

Study limitations include a relatively low initial response fraction, and the exclusion of women with low English language proficiency from participation in the study. Younger women and migrant women of non-English speaking background are under-represented in the sample. It is likely that selection bias may have resulted in underestimation of the prevalence of maternal depression and intimate partner violence. It is also possible that the experiences and health outcomes of migrant women with low English language proficiency may differ from those of migrant women with greater proficiency in English.

While the population-based approach to recruitment via public maternity hospitals resulted in the inclusion of women who had migrated to Australia from many different countries and should be viewed as a strength, it was not possible for us to report data for individual countries of birth. Migrants come to Australia from more than 200 countries [26,27]. Hence, with a total sample of 1507 women, the numbers of migrant women from individual countries are too small for meaningful comparisons, even for the larger source countries. Grouping together migrant women from different countries may mask important differences between women with diverse migration and socio-economic backgrounds, but was unavoidable in the context of the overall design of the study. Building a stronger understanding of the implications of different patterns and contexts for migration for maternal physical and mental health requires the use of tailored sampling and recruitment strategies specifically designed to engage women with diverse cultural backgrounds and low English language proficiency in research [28, 29]. It is also important that we make best use of existing population based data to inform tailoring of maternity and postnatal care to meet the needs of diverse populations.

### Implications for practice

Our findings show that Australian-born and migrant mothers experience significant morbidity in the postnatal period, with many health problems persisting well into the second year after having a baby. The results underline the importance of maternal physical health and mental health surveillance in the postpartum period to ensure that all women have access to appropriate support and information for dealing with common maternal health problems. Importantly, maternal health surveillance needs to be attentive to the high prevalence of intimate partner violence, and potential health consequences for women and their children.

While migrant women experience similar health problems to Australian-born women, they may face additional challenges accessing appropriate care and support related to challenges of cross-cultural communication, availability of interpreters, understandings of health and health care and low health literacy. They are also more likely to experience depressive symptoms and intimate partner violence. An earlier paper from the Maternal Health Study identified that general practitioners and maternal and child health nurses caring for women in the first few months after childbirth are less likely to ask migrant women about their emotional wellbeing or family relationships, than they are to ask Australian-born women [4]. Other studies have shown that migrant women’s experiences of health services and interactions with their caregivers shape their ongoing engagement in health care and help-seeking behaviour [6, 7]. Tailoring care to address these barriers requires health services to move away from a ‘one size fits all’ approach, and adapt service delivery models and approaches to match the needs of diverse populations.
